# Molecular Dissection of Mammalian RNA Polymerase II Transcriptional Termination

**DOI:** 10.1016/j.molcel.2007.12.019

**Published:** 2008-03-14

**Authors:** Steven West, Nicholas J. Proudfoot, Michael J. Dye

**Affiliations:** 1 Sir William Dunn School of Pathology, South Parks Road, Oxford OX1 3RE, UK

**Keywords:** DNA, RNA

## Abstract

Transcriptional termination of mammalian RNA polymerase II (Pol II) is an essential but little-understood step in protein-coding gene expression. Mechanistically, termination by all DNA-dependent RNA polymerases can be reduced to two steps, namely release of the RNA transcript and release of the DNA template. Using a simple nuclear fractionation procedure, we have monitored transcript and template release in the context of both natural and artificial Pol II terminator sequences. We describe the timing and relationship between these events and in so doing establish the roles of the poly(A) signal, cotranscriptional RNA cleavage events, and 5′-3′ exonucleolytic RNA degradation in the mammalian Pol II termination process.

## Introduction

Transcriptional termination (referred to hereafter as termination) is the ultimate step in the transcription cycle, which consists of three phases: transcriptional initiation, which involves the association of RNA polymerase with the DNA template; transcriptional elongation, in which the tight association of polymerase with the DNA template is maintained as the polymerase progresses through the body of the gene; and finally termination, when dissociation of the polymerase and DNA template takes place.

Work chiefly carried out on *E. coli* RNA polymerase has resulted in a detailed description of the mechanism of prokaryotic termination (Henkin, 2000; Nudler and Gottesman, 2002). Furthermore, in eukaryotes, Pol I and Pol III termination are quite thoroughly described (Paule and White, 2000). However, for Pol II, which is responsible for the transcription of all protein-encoding genes, there is still comparatively scant information about the termination process. There are two reasons for this anomaly. First, Pol II termination occurs downstream of the protein-encoding region of the gene so might appear unimportant in terms of gene expression and regulation. Second, Pol II termination studies are technically challenging, as they involve the handling of nascent transcripts.

Since the discovery that a functional poly(A) signal is required for Pol II termination, it has been known that major determinants of termination are the signals that organize processing of the pre-mRNA. This paradoxical situation, in which one process (pre-mRNA processing) appears to control another quite different process (transcription), is now explained by the understanding that transcription and processing of pre-mRNA are intimately linked processes occurring in a complex in which Pol II is just one component (Proudfoot et al., 2002; Rosonina et al., 2006; Zorio and Bentley, 2004). It has also been shown that dedicated termination signals located downstream of the poly(A) signal, in mammalian genes, are required for efficient termination (Dye and Proudfoot, 2001; Gromak et al., 2006; Proudfoot et al., 2002; Tantravahi et al., 1993; West et al., 2006). Possibly the most fully characterized Pol II termination sequence is that located in the 3′ flanking region of the human β-globin gene (Dye and Proudfoot, 2001). Surprisingly, transcripts of the β-globin termination element are cotranscriptionally cleaved by an as-yet-uncharacterized activity termed cotranscriptional cleavage (CoTC). Degradation of the cleaved transcripts by the human nuclear exonuclease Xrn2 is a prerequisite for efficient termination (West et al., 2004). Simultaneous and subsequent studies have underlined the importance of exonucleolytic degradation of pre-mRNA in Pol II termination (Kaneko et al., 2007; Kim et al., 2004). In contrast to the human β-globin gene, experiments in yeast indicate that exonucleolytic degradation initiates on the 3′ product of poly(A) site cleavage rather than on a cleaved termination sequence transcript (Kim et al., 2004). This difference may be because, in yeast, Pol II termination occurs closer to and is more tightly coupled with poly(A) site cleavage. This could be a reflection of the compact nature of the yeast genome. Termination occurs at greater distances downstream of mammalian gene poly(A) sites. We speculate that, in addition to the lower spatial constraints on the boundaries of transcription units in mammalian genomes, the time lag between poly(A) site transcription and subsequent termination might allow a window for processing of complex mammalian pre-mRNAs. For example, in mammalian genes, terminal exon definition might not be completed until some time after the poly(A) site has been transcribed (Bauren et al., 1998; Dye and Proudfoot, 1999).

Pol II termination involves several components: the poly(A) signal, pre-mRNA cleavage (at the poly[A] site or downstream sequences), pre-mRNA degradation, and specific terminator elements. However, a simple model for the mechanism of the termination process is that it involves two steps, namely transcript (RNA) release and template (DNA) release (Proudfoot et al., 2002). Here we describe a direct visualization of the transcript release step in Pol II termination in the context of both natural and artificial Pol II terminator elements. These experiments lead us to a fuller understanding of Pol II termination by illuminating the interrelation of pre-mRNA release and Pol II template release. Moreover, our dissection of the termination process reveals details concerning the roles of the major components involved in Pol II termination.

## Results

**Figure 1:**
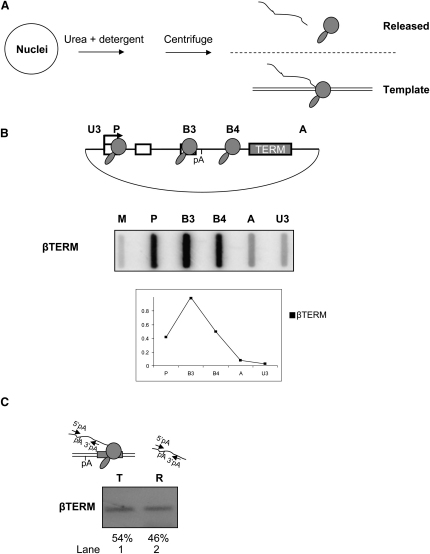
Pol II Releases the DNA Template before Cleavage at the Poly(A) Site (A) Nuclear fractionation procedure. Nuclei were lysed in the presence of urea and detergent. After centrifugation, pre-mRNA (curved line), attached to the DNA template (parallel lines) via Pol II (gray icon), are found in the pellet fraction (template), whereas nucleoplasmic pre-mRNA is found in the supernatant fraction (released). (B) Diagram of βTERM. Transcription start site (arrow), exons (white boxes), the poly(A) signal (pA), and termination element (gray box) are all shown. Data panel shows NRO analysis of βTERM. The regions on the plasmid that were probed for nascent transcription are shown above the relevant slot on the filter and on the plasmid diagram. M is an M13 ssDNA control for background hybridization. The graph shows corrected NRO signals of βTERM where the signal over probe B3 is taken to be 1. The relatively low signal over probe P is due to the fact that transcription initiation does not occur during the NRO reaction. (C) RT-PCR analysis of template (T) and released (R) βTERM pre-mRNA. PCR primers (arrows) are shown in the diagram. Quantitation of PCR products was obtained by real-time PCR.

We have examined the timing of events in pre-mRNA 3′ end processing and Pol II termination using the human β-globin gene as a model system. We employed a nascent pre-mRNA fractionation technique (Wuarin and Schibler, 1994), subsequently modified in our laboratory (Dye et al., 2006), to separate pre-mRNAs on the basis of their association with Pol II. In brief, nuclei are lysed in the presence of urea and a mild detergent. Centrifugation of the lysate pellets pre-mRNA that is linked to chromatin. As we assume that the association of pre-mRNA and Pol II with chromatin is mediated by the association of transcribing Pol II with DNA, we refer to the chromatin pellet as the template fraction. Pre-mRNA that is not associated with chromatin is found in the nucleoplasmic supernatant fraction, termed the released fraction (see diagram, Figure 1A).

We first examined the nuclear distribution of pre-mRNA derived from a construct that supports efficient Pol II termination. Nuclear run-on (NRO) analysis was employed to measure Pol II termination efficiency. HeLa cells were transiently transfected with βTERM, a construct that contains the full β-globin gene and terminator element (TERM), with transcription driven by the HIV-1 promoter activated by cotransfection of a Tat expression vector. NRO analysis of these transfected cells (Figure 1B) shows strong hybridization signals over the genic probes P, B3, and B4 indicating the location of transcribing Pol II. The contrasting low hybridization signals over the extragenic probes, A and U3, are indicative of the absence of transcribing Pol II due to termination in response to the β-globin termination signal (Dye and Proudfoot, 2001). The relative strength of the hybridization signals is shown in the graph below the data panel.

To dissect the termination process at the β-globin terminator, we subjected HeLa cells transfected with βTERM to nuclear fractionation using the technique outlined above (Figure 1A). Following the removal of DNA and protein from the template and released fractions, the remaining RNA was subjected to RT-PCR analysis. Equal amounts of RNA were taken from template and released fractions then reverse transcribed in separate reactions using an oligonucleotide-labeled 3′pA, which is complementary to sequence downstream of the β-globin poly(A) site. The resulting cDNA was then PCR amplified using 3′pA and an oligonucleotide-labeled 5′pA, which is complementary to sequence upstream of the β-globin poly(A) site (see diagram, Figure 1C). Thus, this RT-PCR experiment detects transcripts that are yet to be cleaved at the β-globin poly(A) site. For all RT-PCR experiments in this paper, a parallel experiment was carried out without the reverse transcriptase enzyme to control for DNA contamination (data not shown). The resulting PCR products are shown in Figure 1C. The abundance of PCR product in the template and released PCR reactions is expressed as a percentage of the total PCR product in both fractions. In lane 1, there is a band indicating the presence of 54% of uncleaved pre-mRNA in the template fraction. As we have previously shown that β-globin poly(A) site cleavage is a relatively late event (Dye and Proudfoot, 1999), the appearance of this species is expected, as it represents β-globin pre-mRNA that is in the process of being transcribed. Surprisingly, we detected 46% of this uncleaved β-globin pre-mRNA in the released fraction (lane 2). This indicates that β-globin pre-mRNA, alone or in association with terminated Pol II, has been released from the transcription site before 3′ end processing has taken place. This result indicates that, for a large proportion of β-globin transcripts, 3′ end processing of pre-mRNA does not occur on DNA template-associated Pol II. Furthermore, the detection of a significant level of released pre-mRNA in the presence of efficient Pol II termination indicates that poly(A) site recognition, rather than pre-mRNA cleavage, is the regulator of termination on the β-globin gene. One objection to our interpretation of this data is that we could simply be measuring the abundance of unprocessed transcripts. However, previous studies have shown that virtually 100% of βTERM transcripts are processed at the poly(A) site (Dye and Proudfoot, 2001; West et al., 2004).

**Figure 2:**
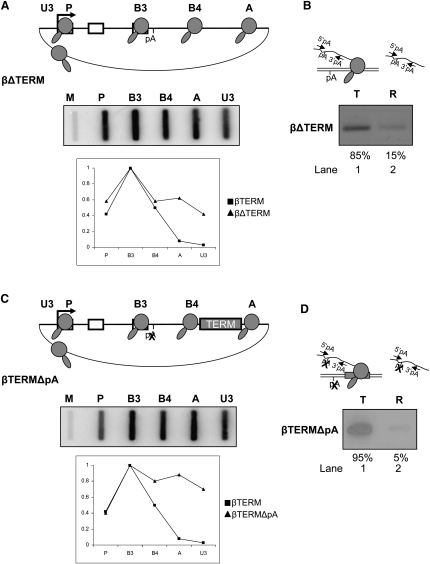
Association of Pre-mRNA with Released Pol II (A) Diagram of βΔTERM. Labeling is as in Figure 1A. Data panel shows NRO analysis of βΔTERM with probes indicated on the diagram and above the filter. The graph compares NRO signals of βTERM and βΔTERM, where the signal over probe B3 is taken to be 1. (B) RT-PCR analysis of template and released βΔTERM pre-mRNA. PCR primers (arrows) are shown in the diagram. Quantitation of PCR products was obtained by real-time PCR. (C) Diagram of βTERMΔpA. X indicates mutation of the poly(A) signal (AATAAA→AATTCC). Data panel shows NRO analysis of βTERMΔpA, with probes indicated on the diagram and above the filter. The graph compares NRO signals of βTERM and βTERMΔpA, where the B3 probe signal was taken as 1. (D) RT-PCR analysis of template and released βTERMΔpA pre-mRNA. PCR primers (arrows) are shown in the diagram. Quantitation of PCR products was obtained by real-time PCR.

To further analyze the relation of transcript release to Pol II termination, we next carried out nuclear fractionation analysis of pre-mRNA from a construct, labeled βΔTERM, a derivative of βTERM from which the terminator element has been deleted (see diagram in Figure 2A). NRO analysis of HeLa cells transiently transfected with βΔTERM shows the effect of the terminator deletion on Pol II transcription (Figure 2A). Hybridization signals over the extragenic probes A and U3 indicate the presence of actively transcribing Pol II that has failed to terminate. Hybridization signals were quantitated and shown alongside hybridization signals from the positive βTERM control in the graph below the data panel.

We next measured the abundance of pre-mRNA derived from βΔTERM in the template and released fractions. RT-PCR analysis (Figure 2B) shows that there is a strong band representing unprocessed pre-mRNA detected in the template fraction (lane 1). Again, these pre-mRNAs are nascent transcripts, yet to be cleaved at the poly(A) site. In lane 2, there is a much weaker band representing the 15% of unprocessed pre-mRNA, which is detected in the released fraction. The appearance of these unprocessed transcripts in the released fraction may result from sporadic termination events occurring at uncharacterized termination signals in the construct. Comparison of this low-level band with the released fraction of βTERM (Figure 1C, lane 2) indicates that Pol II termination is required for release of the unprocessed pre-mRNA transcript to occur. This gives a very strong indication that transcript release occurs concurrently with or shortly after Pol II termination.

We next examined the role of the poly(A) site in transcript release using a construct labeled βTERMΔpA, a derivative of βTERM containing a mutation that functionally inactivates the poly(A) site (see diagram, Figure 2C). NRO analysis of HeLa cells transiently transfected with βTERMΔpA shows strong hybridization signals over the extragenic probes A and U3, indicating transcriptional readthrough (data panel, Figure 2C) and confirming that poly(A) site mutation effectively abolishes Pol II termination. Again, we analyzed the nuclear distribution of βTERMΔpA pre-mRNA transcripts by RT-PCR using the nuclear fractionation protocol (Figure 2D) and show that nearly all (95%) of β-globin pre-mRNA is located in the template fraction (lane 1), with only a small amount released into the nucleoplasmic fraction (lane 2). This experiment shows the requirement of a functional poly(A) site for transcript release to occur. When this result is combined with the above analysis of βTERM and βΔTERM, we can make two conclusions: first, poly(A) site recognition, rather than poly(A) site processing, triggers Pol II termination in the presence of a functional terminator (we return to this point below, Figure 6); second, pre-mRNA release is dependent upon Pol II termination. With reference to the detection of very high levels of βTERMΔpA transcript in the template fraction, it is possible that a contributing factor could be the retention of unprocessed pre-mRNA at transcription sites (Abruzzi et al., 2006; Hilleren et al., 2001).

### Termination at the MAZ4 Sequence Occurs after Poly(A) Site Cleavage

**Figure 3:**
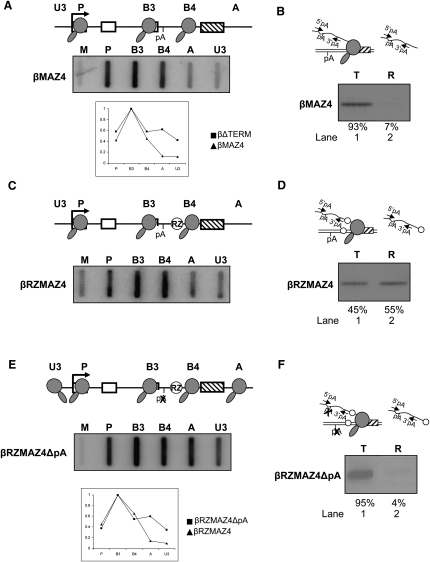
Termination at MAZ Sites Occurs after Poly(A) Site Cleavage (A) Diagram of βMAZ4. The location of the MAZ4 sequence (hatched box) is indicated. Data panel shows NRO analysis of βMAZ4 with probes indicated on the diagram and above the filter. The graph compares NRO signals for βMAZ4 and βΔTERM, where in each case the B3 probe signal is taken to be 1. (B) RT-PCR analysis of template and released βMAZ4 pre-mRNA. PCR primers (arrows) are shown in the diagram. Quantitation of PCR products was obtained by real-time PCR. (C) Diagram of βRZMAZ4. The location of the ribozyme (RZ, circle) is indicated. Data panel shows NRO analysis of βRZMAZ4 with probes indicated on the diagram and above the filter. M is an M13 ssDNA control for background hybridization. (D) RT-PCR analysis of template and released βRZMAZ4 pre-mRNA. PCR primers (arrows) are shown in the diagram. Quantitation of PCR products was obtained by real-time PCR. (E) Diagram of βRZMAZ4ΔpA. X indicates mutation of the poly(A) signal (AATAAA→AATTCC). Data panel shows NRO analysis of βRZMAZ4ΔpA with probes indicated on the diagram and above the filter. (F) RT-PCR analysis of template and released βRZMAZ4ΔpA pre-mRNA. PCR primers (arrows) are shown in the diagram. Quantitation of PCR products was obtained by real-time PCR.

To further analyze the relationship of Pol II termination and transcript release, we used an artificial termination system developed in our laboratory. Positioning of multiple G-rich sequences (MAZ4) downstream of the β-globin poly(A) site enhances termination, possibly through transcriptional pausing of Pol II (Ashfield et al., 1994; Gromak et al., 2006). To confirm that the MAZ4 element promotes termination in our system, we made construct βMAZ4, a derivative of βTERM, in which the β-globin terminator sequence was replaced with the MAZ4 element (see diagram, Figure 3A). We then conducted NRO analysis on HeLa cells transiently transfected with βMAZ4 (Figure 3A). The efficiency of Pol II termination on the βMAZ4 construct is shown by the weak hybridization signals over the extragenic probes A and U3 (Figure 3A) and by comparison with the NRO hybridization signals of βΔTERM in the graph below the data panel. Thus, the MAZ4 element is shown to promote efficient termination.

To dissect the termination process on the βMAZ4 construct, we again conducted nuclear fractionation of transcripts from HeLa cells that had been transiently transfected with βMAZ4. Pre-mRNA from template and released fractions was subjected to RT-PCR analysis (Figure 3B) as described above. In lane 1, there is a strong band showing the high abundance of uncleaved pre-mRNA in the template fraction. In contrast, in lane 2, only a trace amount (7%) of unprocessed pre-mRNA is detected in the released fraction. This result demonstrates that few unprocessed transcripts are released from Pol II terminating at the MAZ4 terminator. As mentioned above, the β-globin terminator element mediates CoTC of nascent transcripts at sites downstream of the poly(A) site. This is an important part of the termination process, as we have previously shown that the 3′ product of CoTC cleavage is a substrate of the exonuclease Xrn2. Degradation of this substrate leads to termination (West et al., 2004). In contrast to the β terminator, the MAZ4 element does not introduce a cleavage into the nascent transcript at downstream sequences. The only cleavage in the transcript, and therefore the entry point for Xrn2, is the poly(A) site. We hypothesize that, with the βMAZ4 construct, unprocessed transcripts are not released because they are processed before termination takes place. To test this possibility, we made a construct labeled βRZMAZ4 (see diagram in Figure 3C) in which a hammerhead ribozyme is positioned just upstream of the MAZ4 sequence in βMAZ4. We and others have previously shown that this RZ cleaves cotranscriptionally and is highly efficient in vivo (Dye et al., 2006; Lacadie et al., 2006; West et al., 2004). NRO analysis of HeLa cells transiently transfected with βRZMAZ4 was used to ascertain whether the introduction of the RZ affected termination at the MAZ4 element. As shown in Figure 3C, NRO analysis of βRZMAZ4 gives an identical transcription profile to that of βMAZ4 (Figure 3A). Therefore, we conclude that RZ cleavage does not affect termination at MAZ sites. As mentioned above, with respect to βTERM, the efficient termination detected on βRZMAZ4 by NRO analysis indicates that the introduction of the RZ has no effect on 3′ end processing efficiency. Quantitative RNA-mapping experiments confirm that the efficiency of 3′ end processing on the βMAZ4 and βRZMAZ4 constructs was identical (data not shown).

We next analyzed the nuclear distribution of βRZMAZ4 pre-mRNAs using RT-PCR analysis as above. The resulting PCR products are displayed in Figure 3D. In lane 1, there is a band showing the presence of uncleaved pre-mRNA in the template fraction. In lane 2, a similar band of slightly higher intensity is detected. The appearance of the uncleaved pre-mRNA transcript in the released fraction indicates that introduction of the RZ in the presence of the MAZ4 terminator causes transcript release. Thus, the combination of the RZ with the MAZ4 sequence mimics the behavior of the β-globin terminator element (Figure 1C). These data underline our conclusion that cleavage of the RNA is an essential step in the termination pathway. It appears that poly(A) site cleavage might only be required for termination in the absence of downstream transcript cleavage events as in βMAZ4. Additional and more rapid cleavage events such as those occurring in β-globin terminator transcripts or RZ in the artificial βRZMAZ4 terminator lead to termination before 3′ end processing. In a natural context, this effect may enhance gene expression by releasing the DNA template from Pol II more quickly so that it is rapidly available for subsequent rounds of transcription.

To examine whether the enhancement of transcript release by the introduction of the RZ was dependent on the presence of a functional poly(A) site, we next mutated the poly(A) signal in βRZMAZ4, forming βRZMAZ4ΔpA. NRO analysis of HeLa cells transiently transfected with βRZMAZ4ΔpA shows strong hybridization signals over the extragenic probes A and U3 (see graph below data panel, Figure 3E), confirming that the poly(A) signal and Pol II termination are disabled on this construct.

We next analyzed the nuclear distribution of βRZMAZ4ΔpA pre-mRNAs by RT-PCR (Figure 3F). In lane 1, there is a band showing the presence of nearly all uncleaved pre-mRNA in the template fraction. In lane 2, a very faint band is detected in the released fraction. Comparison of this result with the nuclear fractionation of βRZMAZ4 shows that mutation of the poly(A) site leads to the retention of pre-mRNAs in the template fraction. This control experiment confirms our previous observation, with the βTERM construct, that transcription of a functional poly(A) signal (poly[A] site recognition) is an absolute prerequisite for both Pol II termination and transcript release. This confirms the causal connection between the two processes and indicates they are completely interrelated, possibly occurring simultaneously.

### Degradation of Pol II-Associated RNA Precedes Termination

**Figure 4:**
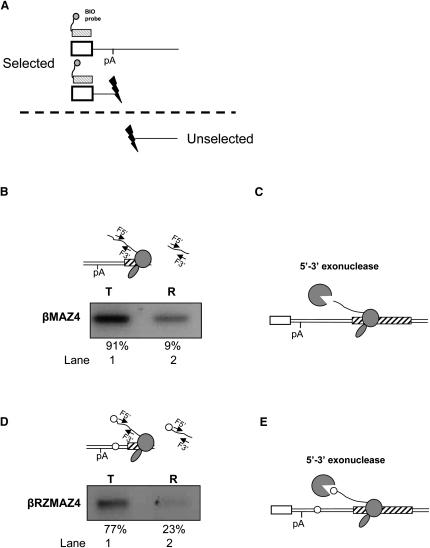
Termination Is Preceded by Degradation of Pol II-Associated RNA (A) Hybrid selection procedure. The biotinylated antisense RNA selection probe (box with tail) hybridizes to exon 3-containing transcripts. After magnetic selection of the hybrids, the 3′ poly(A) site-cleavage product, which does not contain exon 3, remains in the unselected fraction. The lightning bolt denotes cleavage at the poly(A) site. (B) RT-PCR analysis of poly(A) site-cleaved βMAZ4 pre-mRNA in template and released fractions. PCR primers (arrows) are shown in the diagram. Quantitation of PCR products was obtained by real-time PCR. (C) Interpretation of (B). Cotranscriptional poly(A) site cleavage results in a Pol II-associated RNA that is degraded by 5′-3′ exonucleases before termination. (D) RT-PCR analysis of ribozyme-cleaved βRZMAZ4 pre-mRNA in template and released fractions. PCR primers (arrows) are shown in the diagram. Ribozyme is indicated (circle). Quantitation of PCR products was obtained by real-time PCR. (E) Interpretation of (D). Cotranscriptional RZ cleavage results in a Pol II-associated RNA that is degraded by 5′-3′ exonucleases before termination.

We have previously shown, using RNAi to reduce the expression of the human nuclear 5′→3′ exonuclease Xrn2, that efficient Pol II termination on the β-globin gene is dependent on Xrn2 activity (West et al., 2004). In the context of this and previous studies, we hypothesized that cleavage of transcripts by CoTC/RZ promotes termination prior to transcript release by providing an entry site for Xrn2-mediated degradation of the Pol II-associated RNA (the 3′ product of CoTC/RZ cleavage). We therefore employed the nuclear fractionation technique to test this hypothesis. We first analyzed the abundance of the 3′ product of poly(A) site cleavage using the βMAZ4 pre-mRNA where the poly(A) site is the sole entry point for exonuclease. HeLa cells transiently transfected with βMAZ4 were subjected to nuclear fractionation. The βMAZ4 pre-mRNA in the template and released fractions was then subjected to hybrid selection using an antisense biotinylated RNA probe complementary to exon 3 of the β-globin pre-mRNA to remove uncleaved pre-mRNA, as its presence would interfere with the analysis of the cleaved transcripts (see diagram, Figure 4A). The remaining 3′ product of poly(A) site cleavage was then analyzed by RT-PCR. cDNA was made with primer F3′, then PCR amplified with the F3′/F5′ primer pair (see Figure 4B). The strong band in lane 1 represents the 3′ product of poly(A) site cleavage detected in the template fraction (91%). In lane 2, the weaker (9% of total) band represents the same cleaved pre-mRNA species found in the released fraction. This result shows that the majority of the 3′ product of poly(A) site cleavage is associated with the template fraction, indicating that it is attached to Pol II. The low abundance of this transcript in the released fraction indicates that the majority of this cleaved pre-mRNA is not released from the site of transcription. These data indicate a strong correlation between degradation of the 3′ product of poly(A) site cleavage and Pol II template release. This allows us to predict the following sequence of events on the βMAZ4 template: poly(A) site cleavage allows entry of 5′-3′ exonuclease, which degrades the 3′ product of poly(A) site cleavage, thereby mediating Pol II template release (Figure 4C). Our results therefore provide quantitative evidence that degradation of the Pol II-associated downstream product of poly(A) site or CoTC-mediated cleavage is required for Pol II termination.

We repeated the above experiment, this time analyzing the distribution of the 3′ product of RZ cleavage of βRZMAZ4 pre-mRNAs. Following nuclear fractionation of HeLa cells transiently transfected with βRZMAZ4, the 3′ product of RZ cleavage in both template and released fraction was again analyzed by RT-PCR (Figure 4D). As cleavage by the RZ is extremely rapid and highly efficient, there was no need to select uncleaved βRZMAZ4 pre-mRNA away from the template or released pre-mRNA samples. In lane 1, we see the majority (77%) of the 3′ product of RZ cleavage is located in the template fraction, with the remaining 23% in the released fraction (lane 2). The detection of this low level of cleaved pre-mRNA in the released fraction indicates that exonucleolytic degradation, initiating on the 3′ product of RZ cleavage, acts in the same way as degradation initiating on the 3′ product of poly(A) site cleavage to enhance template release (Figure 4E).

In these experiments, we have shown that pre-mRNA degradation initiating after poly(A) site or RZ cleavage largely occurs when Pol II is associated with the DNA template. Degradation of the transcript then leads to template release. This explains how CoTC events in the β-globin terminator transcript lead to Pol II template release before cleavage at the β-globin poly(A) site has occurred.

### The Timing of Transcript Release

**Figure 5:**
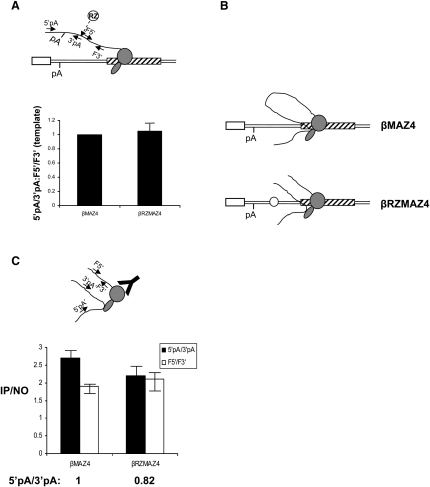
Timing of Pre-mRNA Release (A) Analysis of the template nuclear RNA fraction from HeLa cells transfected with βMAZ4 or βRZMAZ4. Reverse transcription was with F3′ or 3′pA (see diagram). Subsequent real-time PCR was with F5′/F3′ or 5′pA/3′pA, respectively. The ratio of these two species was set at 1 for the βMAZ4 control experiment. Error bars show standard deviation from multiple experiments. (B) Interpretation of (A). 3′ flanking region RNA remains associated with Pol II on βMAZ4 (top). The same is true for βRZMAZ4 despite RZ cleavage breaking the continuity of the transcript. (C) RIP of Pol II-associated RNA from HeLa cells transfected with βMAZ4 or βRZMAZ4. The diagram shows the primers used for reverse transcription (3′pA and F3′) and for subsequent real-time PCR (5′pA/3′pA and F5′/F3′ respectively). The Pol II antibody is also shown (Y). Quantitation is expressed as a ratio of signal obtained in the presence of Pol II antibody to that obtained in a parallel experiment in the absence of any antibody. Consequently, any value higher than 1 represents a specific signal. Error bars show standard deviation from multiple experiments.

We next sought to determine when the released unprocessed pre-mRNAs, observed by nuclear fractionation analysis of βTERM (Figure 1C) and βRZMAZ4 (Figure 3D), are actually detached from the Pol II complex. We reasoned that if pre-mRNA release from Pol II occurred immediately following RZ cleavage of βRZMAZ4 transcripts, then there would be a lower abundance of the upstream transcript compared to the downstream transcript found in the template fraction (see diagram, Figure 5A). Pursuing this rationale, nuclear RNA from HeLa cells transiently transfected with βRZMAZ4 or the control plasmid βMAZ4 was fractionated as described above. The template fractions were reverse transcribed using primer 3′pA to detect the upstream product of RZ cleavage or primer F3′ to detect the downstream product. The resulting cDNAs were then amplified by real-time PCR using the 5′pA/3′pA primer pair or the F5′/F3′ primer pair, respectively (see diagram, Figure 5A). The ratio of the abundance of upstream and downstream sectors of βMAZ4 and βRZMAZ4 3′ flanking region transcripts was calculated, then plotted in the graph shown in Figure 5A. For βMAZ4, we have already shown that 93% of 3′ flanking region transcripts are located in the template fraction (Figure 3A). Therefore, in the control βMAZ4 experiment (lane 1), we have set the ratio of the abundance of upstream and downstream sectors of the 3′ flanking region transcript at 1. Analysis of the template-associated βRZMAZ4 transcripts gives the same result (lane 2). Although the continuity of the upstream and downstream sectors of the βRZMAZ4 3′ flanking region is severed by RZ cleavage, both are equally abundant in the template fraction. Therefore, it appears that transcript cleavage at the RZ does not release the pre-mRNA from the Pol II complex. This indicates that the upstream product of RZ cleavage is tethered to Pol II (see diagram, Figure 5B). This molecular tethering, which has already been described with respect to transcription and pre-mRNA splicing, is not disrupted by the introduction of discontinuity in the RNA chain (Dye et al., 2006).

We wished to provide more direct evidence that the pre-mRNA detected in the released fraction is still tethered to the released (terminated) Pol II complex. RNA immunoprecipitation (RIP) was employed to compare the abundance of unprocessed nascent transcripts associated with Pol II in cells transfected with constructs that did (βRZMAZ4) or did not (βMAZ4) promote transcript release. HeLa cells were transfected with βRZMAZ4 or βMAZ4 and subjected to formaldehyde crosslinking. After cell lysis and sonication of nucleic acids, a Pol II-specific antibody was used to immunoprecipitate Pol II-associated RNA. This RNA was then used as a template for RT-PCR analysis. cDNA synthesis was primed using primers 3′pA and F3′ to detect the 5′ and 3′ sections of the pre-mRNA from both samples. The resulting cDNAs were then real-time PCR amplified using the corresponding 5′pA/3′pA and F5′/F3′ primer pairs (see diagram in Figure 5C). To control for nonspecific immunoprecipitation of RNA, no antibody controls were carried out in parallel and analyzed in the same fashion. As shown graphically in Figure 5C, for βMAZ4 (left hand columns), above-background signals were obtained for both the 5′ and 3′ sectors of the 3′ flanking region, showing their association with Pol II. As both species represent RNA that is connected to Pol II via the continuity of the RNA chain, the ratio of 5′ and 3′ products is set at 1 (indicated below the graph). Analysis of βRZMAZ4 gives a very similar result (right hand columns). It appears that the severance of continuity between the 5′ and 3′ sectors of the βRZMAZ4 3′ flanking region, caused by RZ cleavage, results in a small decrease in the number of unprocessed transcripts (the 5′ sector) associated with Pol II. These data are consistent with the chromatin fractionation results. Strikingly, similar amounts of 5′ sector were immunoprecipitated in both samples in spite of more than 50% of this RNA being present in the released fraction for βRZMAZ4 (Figure 3D). If this released fraction were not associated with Pol II, we would expect that the signal for the 5′ sector of the 3′ flanking region, in the βRZMAZ4 sample, would be ∼50% of that obtained in the βMAZ4 sample. That it is similar (82%) shows that many released pre-mRNAs are associated with Pol II. The fact that a large proportion of the released pre-mRNA detected in the nuclear fractionation experiments above appears to be linked to Pol II provides further evidence for our hypothesis that template release precedes 3′ end processing. Taken together, our results suggest that 3′ end processing of released pre-mRNA occurs on a binary complex of Pol II and RNA that is separate from the DNA template.

### Poly(A) Site Recognition in Transcriptional Termination

**Figure 6:**
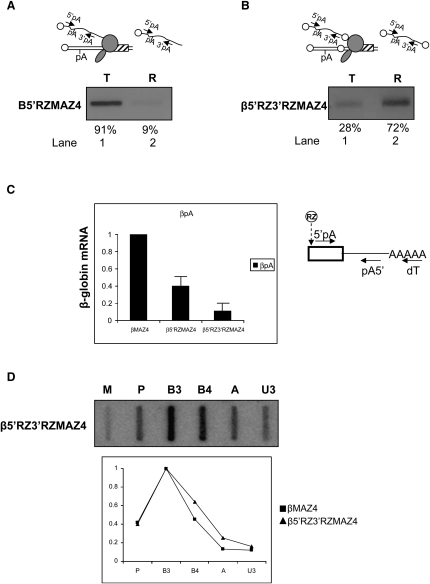
Poly(A) Site Cleavage Is Not Required for Termination (A) RT-PCR analysis of template and released β5′RZMAZ4 pre-mRNA. PCR primers (arrows) are shown in the diagram. RZ (circle) indicates the location of the hammerhead ribozyme. Quantitation of PCR products was obtained by real-time PCR. (B) RT-PCR analysis of template and released β5′RZ3′RZMAZ4 pre-mRNA. PCR primers (arrows) are shown in the diagram. RZ (circle) indicates the location of the two hammerhead ribozymes. Quantitation of PCR products was obtained by real-time PCR. (C) Real-time RT-PCR analysis of polyadenylated β-globin mRNA in cells transfected with βMAZ4, β5′RZMAZ4, or β5′RZ3′RZMAZ4. The value obtained for the βMAZ4-positive control was set at 1. PCR primers (arrows) are shown in the diagram, which depicts the 3′ end of the coding region (white box) until the poly(A) tail. The position of the 5′ RZ is indicated (dotted arrow). Error bars show standard deviation from multiple experiments. (D) NRO analysis of β5′RZ3′RZMAZ4. Probes are indicated on the diagram and above the filter. The graph shows quantitation of NRO signals for β5′RZ3′RZMAZ4 and βMAZ4 where the B3 signal is taken as 1.

We finally wished to examine more closely the role of poly(A) site recognition in the Pol II termination process. To do this, we designed a new construct, labeled β5′RZ3′RZMAZ4, in which ribozymes were positioned both upstream and downstream of the poly(A) site (see diagram, Figure 6B). We envisaged that cotranscriptional RZ cleavage would physically release the poly(A) site-containing fragment of the pre-mRNA from Pol II soon after it had been transcribed. If so, we could monitor any effect transcription of the poly(A) signal has upon Pol II, uncoupled from its role as a pre-mRNA cleavage site. We also made a control construct labeled β5′RZMAZ4, in which only the upstream ribozyme was present (see diagram in Figure 6A).

β5′RZ3′RZMAZ4 and β5′RZMAZ4 were separately transfected into HeLa cells, which were then subjected to nuclear fractionation. Template and released pre-mRNA from each transfection were reverse transcribed using primer 3′pA. The resulting cDNAs were then PCR amplified using the 3′pA/5′pA primer pair to detect pre-mRNA spanning the poly(A) site (see diagram, Figure 6A). For β5′RZMAZ4, a strong band representing the majority (91%) of the uncleaved pre-mRNA was detected in the template fraction (Figure 6A, lane 1). A correspondingly small amount of uncleaved pre-mRNA (9%) was found in the released fraction (lane 2). These results indicate that the presence of the 5′ ribozyme has no significant effect on the retention of the poly(A) signal containing pre-mRNA by transcriptionally engaged Pol II. In contrast, with β5′RZ3′RZMAZ4 (Figure 6B), the transcript distribution is reversed. The majority (∼70%) of the poly(A) signal containing pre-mRNA is found in the released fraction (lane 2), while the remaining 30% of poly(A) signal containing pre-mRNA was found in the template fraction (lane 1). This indicates that RZ cleavage on both sides of the poly(A) signal releases it from Pol II. The PCR product in the template fraction might represent a subpopulation of pre-mRNAs for which binding of poly(A) factors has been more rapid or efficient, so they have remained tethered to the polymerase even after cleavage at the 3′ ribozyme. This experiment shows that the introduction of a single ribozyme upstream of the poly(A) site does not lead to dissociation of the pre-mRNA from Pol II, presumably due to molecular tethering (Dye et al., 2006). However, introduction of the second downstream ribozyme results in dissociation of the majority of the poly(A) site-containing fragments. As this RNA is released, it appears that we have achieved a situation in which we can examine the effect of transcribing the poly(A) site separately from the effect of 3′ end processing on Pol II transcription.

To confirm that we had achieved a real separation of transcriptional and RNA-processing events, we next measured the degree of 3′ end processing of the β5′RZMAZ4 and β5′RZ3′RZMAZ4 pre-mRNAs. As a positive control for efficient 3′ end processing, we used βMAZ4. Equal amounts of nuclear RNA from HeLa cells transiently transfected with either β5′RZMAZ4, β5′RZ3′RZMAZ4, or βMAZ4 were reverse transcribed using oligo-dT. The resulting cDNAs were then real-time PCR amplified with the pA5′/5′pA primer pair (Figure 6C). Column 1 shows the level of nuclear mRNA resulting from expression of βMAZ4, which we arbitrarily set at 1. In column 2, we see a reduced (40%) level of cleaved and polyadenylated RNA from β5′RZMAZ4. We suggest that this reduction is due to the instability of this species caused by RZ cleavage, which renders it susceptible to exonucleolytic degradation. In the β5′RZ3′RZMAZ4 sample (column 3), we see that cleavage of the pre-mRNA upstream and downstream of the poly(A) signal further reduces the level of cleaved and polyadenylated RNA to about 10% of the βMAZ4 sample. The stability of polyadenylated RNA from β5′RZ3′RZMAZ4 and β5′RZMAZ4 would be expected to be similar, since the RNAs would have the same 5′ (RZ-generated) and 3′ (polyadenylated) ends. Thus, comparison of β5′RZMAZ4 with β5′RZ3′RZMAZ4 shows that RZ cleavage either side of the poly(A) site releases the RNA from Pol II before 3′ end processing can take place. Therefore, for the majority of transcripts (90%), we have uncoupled poly(A) site transcription (recognition) from poly(A) site processing.

We finally tested if the poly(A) signal flanked by RZs, in β5′RZ3′RZMAZ4, was still able to function as a transcriptional termination signal using NRO analysis. Examination of the transcription profile of β5′RZ3′RZMAZ4 (Figure 6D) shows strong hybridization signals over the genic probes, P, B3, and B4, and background level hybridization signals over the extragenic probes, A and U3, equivalent to the positive termination control (βMAZ4, see Figure 3A). Therefore, although dissociation of the poly(A) signal from Pol II impairs 3′ end processing, termination is unaffected. This indicates that, even though the association of the poly(A) site transcript with Pol II is brief, it is long enough for the transmission of the signal that the 3′ end of the gene has been reached. These data confirm the conclusion we have made from much of the data above, that transcription of the poly(A) signal is sufficient to render Pol II termination competent. We suggest that poly(A) signal recognition plays the primary role of the poly(A) signal in Pol II transcription termination. It appears that cleavage at the poly(A) signal performs a secondary role in termination, which is only required in the absence of downstream transcript cleavage events, such as those that occur in transcripts of the β-globin terminator sequence.

## Discussion

**Figure 7:**
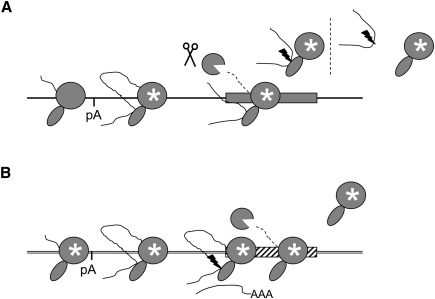
The Sequence of Events in Pol II Transcription Termination (A) The human β-globin terminator (or βRZMAZ4): transcription of the poly(A) signal (pA) switches Pol II (gray icons) to a termination prone form (white star). CoTC of the terminator region transcript, denoted by the scissors, is followed by degradation of the Pol II-associated pre-mRNA (dashed line), which leads to template release. For a significant proportion of pre-mRNA, poly(A) site cleavage (lightning bolt) occurs on Pol II that is dissociated from the DNA template, i.e., in a binary complex. Though our results do not favor the hypothesis, it is possible that cleavage of some released pre-mRNA occurs off Pol II. (B) The MAZ4 terminator: transcription of the poly(A) signal switches Pol II to a termination-prone form. Cleavage at the poly(A) site (lightning bolt) is followed by degradation of the Pol II-associated pre-mRNA (dashed line), which leads to template release. The majority of 3′ end processing occurs while Pol II is associated with the DNA template, i.e., in the ternary complex. The location of the MAZ4 sequence (hatched box) is indicated.

Since the original finding that the poly(A) signal is required for Pol II termination (Whitelaw and Proudfoot, 1986), subsequent studies have revealed roles for other RNA sequences, DNA sequences, and *trans*-acting protein factors in this process (Proudfoot et al., 2002). It has been unclear how interaction of these components brings about Pol II termination. We can now present a model showing the sequence of events in the Pol II termination process (Figure 7).

For the human β-globin gene (Figure 7A), transcription of the poly(A) signal renders Pol II termination competent (white star). This occurs very rapidly, as shown in our final experiment (Figure 6). Next, Pol II synthesizes the β-globin terminator transcript, which is cotranscriptionally cleaved (CoTC; Dye and Proudfoot [2001]), resulting in the exposure of an unprotected 5′ end, a substrate for 5′-3′ exonuclease. Degradation of this Pol II-associated transcript precedes template release (Kaneko et al., 2007; West et al., 2004; Kim et al., 2004). This model was validated using βRZMAZ4, which essentially recapitulates events on the β-globin terminator in a manner that is more amenable to study.

Pol II termination on genes where there is no CoTC-termination element, and therefore no exonuclease entry site downstream of the poly(A) site, involves a different sequence of events (Figure 7B). Our results, using the artificial βMAZ4 terminator construct, show that poly(A) site recognition causes Pol II to become termination competent. We show that poly(A) site cleavage occurs while Pol II is bound to DNA. Exonucleolytic degradation of the Pol II-associated downstream product of poly(A) site cleavage again precedes Pol II template release.

Most interestingly, our data show that Pol II termination on the human β-globin gene occurs prior to cleavage at the poly(A) site, for a significant fraction of the pre-mRNA. For these pre-mRNAs, poly(A) site cleavage could occur independently of the Pol II complex or, alternatively, together with the polymerase as part of a binary complex (Figure 7A). Our results indicate that, in many cases, poly(A) site cleavage of human β-globin pre-mRNA occurs on a binary complex of pre-mRNA and Pol II that has released from the DNA template, rather than a ternary complex composed of pre-mRNA, Pol II, and template DNA.

We have resolved a number of questions concerning Pol II termination. We have shown that poly(A) site recognition occurs rapidly and is essential to render Pol II termination competent. We also show, by RNA-mapping experiments, that 5′-3′ exonucleolytic degradation of cleaved pre-mRNA precedes Pol II template release. However, cleavage at the poly(A) site, which allows an entry point for 5′-3′ exonuclease, is only required in the absence of a downstream transcript cleavage event. We have demonstrated that Pol II termination occurs by a combination of the two original models for the poly(A) site dependence of Pol II termination, which were (1) that Pol II termination occurs in response to changes in the Pol II complex brought about by transcription of the poly(A) signal (Logan et al., 1987), and (2) that Pol II termination occurs in response to degradation of the 3′ flanking region transcript, the “Torpedo” model (Connelly and Manley, 1988). Effectively, our findings show how these two original termination models merge and offer a full description of the order of events in mammalian Pol II termination.

## Experimental Procedures

### PCR Primers

A list of oligonucleotide sequences used as PCR primers is given in the Supplemental Data available online.

### Plasmid Constructions

βTERM, βΔTERM, and βTERMΔpA refer to the previously described βΔ5-7, βΔ5-10, and βΔ5-7p(A)mut plasmids (Dye and Proudfoot, 2001). βMAZ4 corresponds to the previously described MAZ_4_ construct (Gromak et al., 2006). VA and Tat plasmids have also been described previously (Dye and Proudfoot, 1999). βRZMAZ4 was made by insertion of the annealed HHF/HHR oligo pair into plasmids prepared by PCR amplification of βMAZ4 using oligos F5′ and F3′. βRZMAZ4ΔpA was made by ligation of the product of PCR amplification of βRZMAZ4 with primers pA5′ and ΔpAF. β5′RZMAZ4 was made by inserting the annealed HHF/HHR oligos into βMAZ4, digested with BstX1. β5′RZ3′RZMAZ4 was made by inserting the annealed HHF/HHR oligos into a vector prepared by PCR of β5′RZMAZ4 with the 3′pA/F5′ primer pair.

### Transfection Procedure

Transient transfection of HeLa cells was performed as described previously (Dye et al., 2006).

### Nuclear RNA Fractionation

Nuclear RNA fractionation was performed as described (Dye et al., 2006).

### NRO Analysis and Single-Stranded Probes

NRO analysis and single-stranded M13 probes used are as described previously (Dye and Proudfoot, 1999; West et al., 2004). Quantitation of NRO hybridization signals by phosphoimager analysis is based on the average of multiple experiments after subtraction of background signal, shown by probe M.

### Hybrid Selection of Transcripts

The plasmid template and in vitro transcription conditions used for synthesizing the exon 3 selection probe and the protocol for magnetic selection of RNA hybrids have been described previously (Dye and Proudfoot, 1999).

### RT-PCR

cDNA was synthesized using SuperScript III (Invitrogen). DNA amplification was performed using Taq DNA polymerase or, when conducting real-time PCR, using a QIAGEN SYBR Green kit. All quantitations of real-time PCR products are based on the average of multiple experiments.

### RNA Immunoprecipitation

In brief, the protocol of Niranjanakumari et al. (2002) was used for crosslinking, sonication, and antibody pull-down. However, we obtained better results by performing the subsequent wash step as described by Kadener et al. (2002). The Pol II N-20 antibody (Santa Cruz) was used for immunoprecipitation.

## Supplemental Data

Document S1. One Table
