# Supplementary material for: Molecular Dissection of Mammalian RNA Polymerase II Transcriptional Termination
Source: Mol Cell. 2008 Mar 14;29(5):600–10. doi: 10.1016/j.molcel.2007.12.019 (PMC2288634; doi:10.1016/j.molcel.2007.12.019)
Supplement: Document S1. One Table [file mmc1.pdf]

Molecular Cell, Volume *29*

Supplemental Data

Molecular Dissection of Mammalian

RNA Polymerase II Transcriptional Termination

Steven West, Nicholas J. Proudfoot, and Michael J. Dye

Table S1.

Oligonucleotide sequences (5'→3')

**5'pA**	GGGATATTATGAAGGGCCTTGAC
**3'pA**	GAACTAGCTCTTCATTTCTTTATG
**F5'**	CCTTGGGAAAATACACTATATC
**VF**	CAGGAAACTATTACTCAAAGGGTA
**F3'**	TTGAATCCTTTTCTGAGGGATG
**pA5'**	AATCCAGATGCTCAAGGCC
**HHF**	CCTGTCACCGGATGTGTTTTCCGGTCTGATGAGTCCGTGAGGAC GAAACAGG
**HHR**	CCTGTTTCGTCCTCACGGACTCATCAGACCGGAAAACACATCCG GTGACAGG
**HHmF**	GTGTTTTCCGGTCTCATGAGTCCGTGAG
**HHmR**	CTCACGGACTCATGAGACCGGAAAACAC
**ΔpAF**	CTGCCGAATTCAAAACATTTATTTTC
